# Risk perception and influenza vaccine hesitancy among university students in Saudi Arabia

**DOI:** 10.3389/fphar.2026.1747976

**Published:** 2026-01-19

**Authors:** Faris S. Alnezary, Rama A. Alhejaili, Alaa A. Alayashi, Dina S. Alnizari, Nada S. Bin Mirdhah, Shaden K. Alrays, Saad Alobid, Fahad Alzahrani, Haifa A. Fadil, Waad Alrohily, Abrar K. Thabit, Mansour A. Mahmoud, Masaad S. Almutairi

**Affiliations:** 1 Department of Pharmacy Practice, College of Pharmacy, Taibah University, Madinah, Saudi Arabia; 2 Department of Pharmacology, College of Pharmacy, King Saud University, Riyadh, Saudi Arabia; 3 Department of Pharmacy Practice, Faculty of Pharmacy, King Abdulaziz University, Jeddah, Saudi Arabia; 4 Department of Pharmacy Practice, College of Pharmacy, Qassim University, Qassim, Saudi Arabia

**Keywords:** acceptance, vaccines, vaccine hesitancy, influenza vaccines, Saudi Arabia, university students

## Abstract

**Introduction:**

University campuses are high-risk environments for influenza transmission, yet vaccine coverage among students often remains suboptimal. This study aimed to identify the determinants of vaccine hesitancy among university students in Saudi Arabia using the Health Belief Model (HBM) framework.

**Method:**

A cross-sectional study was conducted between January and September 2024. Data were collected via a validated online Arabic questionnaire assessing HBM constructs—susceptibility, severity, benefits, and barriers—alongside sociodemographic variables. Ethical approval was obtained for human participation.

**Results:**

Of the 450 university students surveyed, 62.4% reported encountering significant barriers to vaccination. Despite a high recognition of vaccine benefits (66.7%), this awareness did not correlate with higher intention to vaccinate after adjusting for sociodemographic factors. Students perceiving fewer barriers exhibited substantially higher acceptance rates compared to those perceiving high barriers (60% vs. 27.8%). Furthermore, female students were nearly twice as likely to report moderate barriers (aOR = 1.994) compared to males, while healthcare students were significantly less likely to perceive such obstacles (aOR = 0.654). Students with chronic conditions also demonstrated higher vaccine acceptance (44.1%) compared to their healthy peers (20.2%).

**Conclusion:**

Influenza vaccine uptake among Saudi university students is hindered primarily by perceived barriers rather than a lack of awareness regarding vaccine benefits. Public health strategies on campuses should shift focus from simply emphasizing advantages to actively mitigating logistical and misconception-based obstacles, particularly targeting non-healthcare disciplines and female students.

## Introduction

Influenza is a highly contagious respiratory illness with rapidly changing viral strains and high transmission rates, especially in crowded settings such as universities (Borkenhagen et al., 2021; Javanian et al., 2021; Virk et al., 2017). Although generally self-limiting, it can lead to substantial morbidity and mortality in vulnerable groups, including the elderly, pregnant women, and immunocompromised individuals (Li et al., 2021; Chen et al., 2021). Globally, seasonal influenza affects about one billion people each year, with 3–5 million severe cases and up to 650,000 respiratory deaths (Lafond et al., 2021; Paget et al., 2023; Lampejo, 2022). And remains a significant cause of lower respiratory complications in young children (Wang et al., 2020).

Annual influenza vaccination is required due to continual viral mutations (Houser and Subbarao, 2015), yet vaccine hesitancy remains a major barrier. Common obstacles include limited knowledge, misconceptions, and concerns about vaccine effectiveness and safety, all of which reduce willingness to vaccinate (Ahmed et al., 2023; Hwang, 2020; Suryadevara, 2021). Similar patterns appear in Saudi Arabia, where additional misconceptions, such as the belief that the vaccine can cause influenza, further hinder uptake (Almansour et al., 2022; Mallhi et al., 2022; Haridi et al., 2017; Abalkhail et al., 2017). Socioeconomic factors present a complex landscape, as income levels can influence vaccine acceptance both positively and negatively (Al-Mohaithef and Padhi, 2020). Socioeconomic and educational differences also contribute to variability in acceptance (Al-Mohaithef and Padhi, 2020). Although the Saudi Ministry of Health provides free vaccines and conducts extensive awareness campaigns, hesitancy remains high despite strong evidence supporting vaccine safety and effectiveness (Alhatim et al., 2022; Alabbad et al., 2018; Fayed et al., 2021; Alghalyini et al., 2023).

University students in Saudi Arabia represent a high-risk group where behavioral barriers intersect with the challenges of densely populated living and learning environments.

(Sheldon et al., 2021; Nichol et al., 2005; Alhatim et al., 2022). Many young adults underestimate their risk, which affects vaccine uptake despite the Ministry of Health offering free seasonal influenza vaccines (Siegel et al., 2007). However, limited research has applied the Health Belief Model to understand the psychological factors shaping hesitancy in this population. This study aims to examine how perceived risk relates to influenza vaccine hesitancy among university students in Saudi Arabia, with the goal of informing strategies to improve vaccination coverage and public health outcomes.

## Methods

### Study design and participants

A cross-sectional study was conducted utilizing the Health Belief Model to examine vaccine hesitancy among university students in Saudi Arabia. The study was conducted from January 2024 to September 2024 and included a sample of university students. Vaccine acceptance was defined as self-reported receipt of the influenza vaccine within the previous year. Participants were recruited via major social media platforms, including Twitter, WhatsApp, and Telegram, employing a convenience sampling approach.

### Survey development and data collection

The questionnaire was developed utilizing a structured survey instrument modified from previous studies and subsequently translated from English to Arabic through the back-translation methodology (Du et al., 2022; Hu et al., 2017). This approach ensures accuracy and clarity in the translation by first translating the survey from English to Arabic, followed by re-translating it back into English by a separate translator. The final survey was pilot tested with a sample of 15 students to assess clarity and comprehensibility. The Cronbach’s alpha for all HBM construct combined was 0.78. Cronbach’s alpha for each construct was also tested and it was found to be 0.65 for perceived susceptibility, 0.51 for perceived severity, 0.61 for perceived barriers and 0.88 for perceived benefits. The final version comprised 32 closed-ended questions, organized into five distinct sections, and was uploaded to Google Forms.

The first section solicited sociodemographic information, which included age, sex, residence (urban/rural), marital status, educational attainment of both respondents and heads of household, as well as income categories that delineate non-poor status, according to the national poverty line figures for the years 2017–2020, adjusted to reflect changes resulting from the economic crisis. This section also included data from the National Income Consumption Survey Decile on *per capita* monthly expenditures and a query on the respondent’s nationality.

The subsequent four sections were constructed based on the Health Belief Model (HBM) constructs, evaluating perceived susceptibility to influenza and adverse reactions to vaccination, as well as the perceived severity of both (Rosenstock et al., 1988). Additionally, the sections addressed respondents’ intentions regarding vaccination, their perceived barriers to vaccination, and the anticipated benefits of vaccination. All questions related to the HBM were rated on a five-point Likert scale. Each HBM construct consisted of 5 questions with a total score of 25. The total score was recoded into 3 categories (Low; total score of 1–10), (moderate; total score of 11–17) and high; (total score of 18–25). Participants were recruited via major social media platforms, including Twitter, WhatsApp, and Telegram, employing a convenience sampling approach.

### Ethical approval and data confidentiality

The ethical approval for the study was obtained from the Research Ethics Committee at the College of Pharmacy, Taibah University (Reference No: COPTU-REC-91–20240303). All data collected during the study was treated with the highest level of confidentiality. Participant identities and responses were kept anonymous and securely stored, with access restricted only to authorized personnel involved in the research. No personally identifiable information was shared or published without consent from the participants. All data were retained in compliance with relevant privacy laws and ethical standards.

### Data analysis

Descriptive statistics were presented as means with standard deviations (SD) for continuous variables and as percentages (%) for categorical data. Normality testing was conducted for the HBM construct scores, and the results indicated that the data were not normally distributed. Pearson’s χ^2^ test and Fisher’s exact test were employed, as appropriate, to examine the association and compare the demographic characteristics of participants with vaccine acceptance. Both crude and adjusted odds ratios (aORs) of vaccine acceptance across various risk-perception groups were estimated using univariate and multivariable binary logistic regression models. Sensitivity analyses were conducted by fitting multiple models to assess the robustness of our estimates. Model A consisted of a binary univariate logistic regression model with vaccine acceptance as the dependent variable and each risk-perception category entered individually as the independent variable. Model B was a multivariable binary logistic regression model with vaccine acceptance as the dependent variable and all risk-perception categories included simultaneously as independent variables. Multiple binary logistic regression subgroup analysis was also performed to examine the relationship between moderate barriers (compared with low and high barriers combined) as the dependent variable and various sociodemographic characteristics as independent variables. A *P* value of <0.05 was considered significant. Statistical analyses were executed using IBM SPSS Statistics (version 26, IBM Corp., Armonk, NY, United States).

### Sample size calculation

A minimum sample size of 385 was determined using the Raosoft® online calculator (Raosoft, 2004), based on the following parameters: a 5% margin of error, a 95% confidence interval, a target population of 1.5 million, and an estimated response distribution of 50%. To account for potential dropouts, an additional 15% was added, resulting in a revised minimum sample size of 443 participants.

## Results

A total of 450 university students have completed the questionnaire. The detailed results are presented in Table 1. The mean for perceived susceptibility was 3.06 (SD = 0.54); most students (78.4%) showed a moderate level of susceptibility. For perceived severity, the mean score was 3.43 (SD = 0.57), with almost half of the participants (47.6%) considered the risk to be high. Perceived barriers mean score was 3.73 (SD = 0.67), which means that 62.4% of students reported important barriers to vaccination. The average of perceived benefits was 3.74 (SD = 0.82) with most students perceiving high (66.7%), moderate (28.4%), or low (4.9%) benefits.

**TABLE 1:** Risk perception of 450 university students.

Risk perception	Mean ± SD or N (%)
Perceived susceptibility	3.06 ± 0.54
Low	19 (4.2)
Moderate	353 (78.4)
High	78 (17.3)
Perceived severity	3.43 ± 0.57
Low	3 (0.7)
Moderate	233 (51.8)
High	214 (47.6)
Perceived barriers	3.73 ± 0.67
Low	5 (1.1)
Moderate	164 (36.4)
High	281 (62.4)
Perceived benefit	3.74 ± 0.82
Low	22 (4.9)
Moderate	128 (28.4)
High	300 (66.7)

**FIGURE 1:**
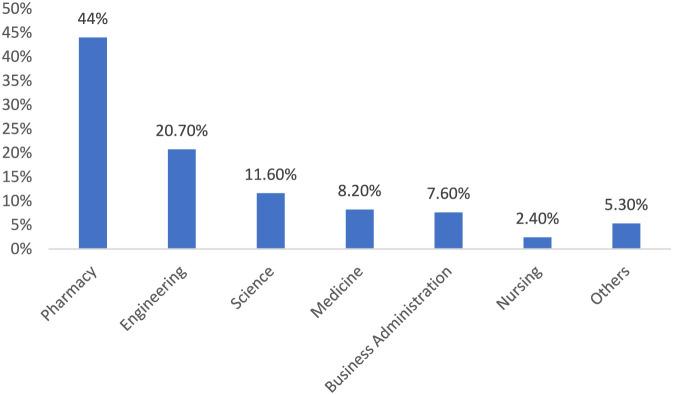
The percentage distribution of participants across different colleges.

Among the studied population 99 (22%) accepted flu vaccine (Table 2). The acceptance rate was significantly higher (44.1%) in students suffering from chronic conditions compared to students without such conditions (20.2%), p = 0.002. However, the effect size was small (Cramér’s V = 0.153). Furthermore, among those who perceived low barriers to vaccination, the acceptance rate was higher (60%) than those who perceived high barriers (27.8%) and low barriers (11%), with a p-value of <0.001. The effect size was small to moderate (Cramér’s V = 0.217). Finally, the acceptance rate from students who acknowledged the low benefits of the flu vaccine (36.4%) was higher than high (24.0%) and moderate (14.8%) benefits with p = 0.029. However, the effect size was small (Cramér’s V = 0.126). Figure 1 represents students’ participation from different colleges. The result indicates that most of the students were from pharmacy colleges (44%), followed by those from engineering colleges (20.7%).

**TABLE 2:** Flu vaccination acceptance among university students.

Characteristics	N	Receiving flu vaccine (%)	P value
Total	450	99 (22)	​
Gender	​	​	0.182^a^
Male	139	36 (25.9)	​
Female	311	63 (20.3)	​
Age	​	​	0.595^a^
17–20	176	41 (23.3)	​
21 and above	274	58 (21.2)	​
Province	​	​	0.331^b^
Central	62	9 (14.5)	​
Eastern	34	10 (29.4)	​
Western	341	77 (22.6)	​
Southern and northern	12	3 (23.1)	​
Social history	​	​	0.087^a^
Single	435	93 (21.4)	​
Married	15	6 (40)	​
Academic year	​	​	0.743^a^
First	74	21 (28.4)	​
Second	98	18 (18.4)	​
Third	87	21 (24.1)	​
Fourth	70	13 (18.6)	​
Fifth	77	17 (22.1)	​
Sixth	44	9 (20.5)	​
Healthcare college	​	​	0.065^a^
Yes	264	50 (18.9)	​
No	186	49 (26.3)	​
Family income	​	​	0.535^a^
<5,000 riyals	74	21 (28.4)	​
5,000–10,000	101	22 (21.8)	​
11,000–15000	86	18 (20.9)	​
>15,000	189	38 (20.1)	​
Nationality	​	​	0.237^b^
Saudi	440	95 (21.6)	​
Non-saudi	10	4 (40)	​
Chronic condition	​	​	0.002^a^
Yes	34	15 (44.1)	​
No	416	84 (20.2)	​
Risk perception			
Perceived susceptibility	​	​	0.140^b^
Low	19	1 (5.3)	​
Moderate	353	83 (23.5)	​
High	78	15 (19.2)	​
Perceived severity	​	​	0.193^b^
Low	3	2 (66.7)	​
Moderate	233	50 (21.5)	​
High	214	47 (22)	​
Perceived barriers	​	​	<0.001^b^
Low	5	3 (60)	​
Moderate	164	18 (11)	​
High	281	78 (27.8)	​
Perceived benefit	​	​	0.029^a^
Low	22	8 (36.4)	​
Moderate	128	19 (14.8)	​
High	300	72 (24)	​

^a^ Chi square.

^b^ Fishers exact test.

Based on the three factors identified in the feature selection, the logistic regression models were established as model A and model B, respectively (Table 3). In model A, not adjusting for other factors, moderate perceived barrier (P < 0.001) and moderate perceived benefit (P = 0.036) were associated with the vaccine acceptance variable. Perceived benefit was not significantly associated with vaccine acceptance after adjustment for sociodemographic characteristics (model B). On the contrary, a moderate perceived barrier found a good association with vaccine acceptance (P < 0.010).

**TABLE 3:** Association between risk perception and vaccine acceptance.

Variables	Model A crude odds ratio (95% CI)	P value	Model B adjusted odds ratio (95% CI)	P value
Perceived susceptibility				
Low	0.233 (0.029–1.88)	0.173	0.111 (0.011–1.092)	0.059
Moderate	1.29 (0.698–2.387)	0.415	1.200 (0.624–2.305)	0.585
High	Reference	​	Reference	​
Perceived severity				
Low	Reference	​	Reference	​
Moderate	0.137 (0.012–1.537)	0.107	0.237 (0.019–2.953)	0.263
High	0.141 (0.012–1.586)	0.113	0.243 (0.019–3.059)	0.273
Perceived barriers				
Low	Reference	​	Reference	​
Moderate	0.082 (0.013–0.525)	0.008	0.059 (0.007–0.515)	0.010
High	0.256 (0.042–1.562)	0.140	0.168 (0.020–1.408)	0.100
Perceived benefit				
Low	1.810 (0.730–4.487)	0.201	2.586 (0.813–8.226)	0.107
Moderate	0.552 (0.317–0.961)	0.036	0.624 (0.339–1.149)	0.130
High	Reference	​	Reference	​

Moderate perceived barriers were most strongly associated with vaccine acceptance. To investigate this association, we performed multiple logistic regression analyses controlled for sociodemographic variables. Results indicated that females were more likely than males to experience moderate barriers with adjusted odds ratio (aOR) = 1.994 (95% CI: 1.267–3.138). On the other hand, students from healthcare colleges tended to have moderate barriers less often (aOR) = 0.654 (95% CI: 0.428–0.998) (Table 4).

**TABLE 4:** Association of moderate perceived barriers with sociodemographic characteristics.

Characteristics	Adjusted odds ratio (95% confidence interval)	P value
Gender		
Female	1.950 (1.240–3.063)	0.004
Male	Reference	​
Age		
17–20	1.303 (0.690–2.462)	0.415
21 and above	Reference	​
Province		
Central	1.026 (0.273–3.862)	0.970
Eastern	0.614 (0.143–2.85)	0.497
Western	1.162 (0.338–3.997)	0.812
Southern and northern	Reference	​
Social		
Single	2.090 (0.621–7.031)	0.234
Married	Reference	​
Academic year		
First	0.547 (0.203–1.476)	0.234
Second	0.661 (0.262–1.669)	0.381
Third	0.891 (0.394–2.019)	0.783
Fourth	0.869 (0.385–1.961)	0.736
Fifth	0.851 (0.382–1.898)	0.694
Sixth	Reference	​
Healthcare college		
Yes	1.563 (1.025–2.383)	0.038
No	Reference	​
Family income		
<5,000 saudi riyals	0.835 (0.464–1.501)	0.547
5,000–10,000 saudi riyals	1.008 (0.604–1.682)	0.975
11,000–15000 saudi riyals	1.060 (0.618–1.819)	0.832
>15,000 saudi riyals	Reference	​

## Discussion

This national online survey presents novel findings that contribute to the understanding of risk perception theory and influenza vaccine hesitancy among university students in Saudi Arabia. Our results indicate that vaccine acceptance is influenced by a multifactorial interplay, with perceived barriers identified as the strongest predictor. Specifically, a majority of respondents reported high barriers to vaccination; conversely, individuals who perceived low barriers demonstrated significantly higher rates of acceptance. A noteworthy paradox emerged in that, despite high perceived benefits, this did not correlate with increased vaccine acceptance rates when sociodemographic factors were accounted for.

While university students are typically at lower risk for mortality, the relevance of these benefits lies in mitigating morbidity and preserving academic continuity. As demonstrated by Nichol et al., influenza significantly impairs university students’ academic performance and increases healthcare resource utilization (Nichol et al., 2005). Furthermore, high coverage in this group is essential for reducing community transmission to vulnerable populations. However, our data indicates that students are already largely aware of these advantages; therefore, this finding suggests that enhancing perceived benefits may be less critical than reducing perceived barriers in the formulation of effective intervention strategies. This lack of predictive power can be attributed to the “overriding” effect of barriers. Within the HBM framework, while perceived benefits provide the motivation to act, perceived barriers function as the immediate impediments to that action. Since substantial proportion of our sample reported high barriers, these practical and psychological hurdles likely negate the positive influence of benefit awareness, effectively severing the link between knowledge and actual behavior (Liu et al., 2025).

The findings of this study both align with and diverge from existing research regarding attitudes toward influenza vaccination and the associated barriers at the university students’ level. The observed contradiction between the perceived high barriers (62.4%) and the high benefit scores (66.7%) in our study corresponds with similar patterns identified in studies conducted across various countries. Furthermore, multiple studies have identified barriers related to safety (Mallhi et al., 2022; Benjamin and Bahr, 2016), knowledge (Hashmi et al., 2016; Su and Chen, 2023), and time/convenience (Ryan et al., 2019; Abushouk et al., 2023). The consistency of this factor suggests that the impediments to vaccination are not confined to individual institutions but rather indicate broader issues affecting the student population.

Healthcare education has emerged as a significant factor across various studies. The observed lower perceived barriers among healthcare students aligns with findings from multiple prior studies, which demonstrate that healthcare students exhibit markedly higher vaccine acceptance, as assessed through knowledge scores (Mallhi et al., 2022; Al Nufaiei et al., 2023) or vaccination rates (Ryan et al., 2019). This trend may imply that formal health education fosters a more comprehensive understanding of the benefits and risks associated with vaccines. However, a recent study conducted by Zou et al. in China indicated that medical students displayed greater hesitation compared to their social science counterparts, indicating possible differences not only in health education between countries but also in cultural aspects of healthcare education itself (Zou et al., 2023). These variations highlight the necessity of considering cultural and contextual factors when formulating educational interventions.

Variations in the relationship between gender and vaccine perceptions across different studies have been observed. While prior research has produced conflicting results, our study identified an increased likelihood of females experiencing moderate barriers to vaccination. Some studies indicated that females were more inclined to support vaccination (Abushouk et al., 2023; Silva et al., 2021), whereas others found no significant gender differences (Al Nufaiei et al., 2023; Bednarczyk et al., 2015), and additional studies reported contrary trends (Zou et al., 2023). This variability suggests that the influence of gender on vaccine acceptance is highly context-dependent. In the specific setting of Saudi Arabia, the increased perception of barriers among females may reflect distinct logistical and social challenges documented in regional literature. Previous studies indicate that women in Saudi Arabia often face greater difficulties accessing healthcare facilities due to reliance on male family members for transportation and competing domestic responsibilities (Mobaraki and Söderfeldt, 2010). Furthermore, women often exhibit higher health anxiety and concern regarding vaccine side effects compared to men, which contributes to the ‘psychological’ aspect of the HBM barriers construct (Fisher et al., 2020).

The role of health awareness and risk perception in relation to vaccination acceptance emerged as a prominent theme; however, significant variability was observed. Our study indicated notably higher acceptance rates among students with chronic conditions, although the specific relationship was not directly investigated in the studies under comparison. Notably, Bednarczyk, et al. showed that healthy status was identified as one of the contributing factors to vaccine hesitancy, with 29% of unvaccinated students reporting the belief that “I do not need it because I am healthy” (Bednarczyk et al., 2015). Furthermore, the study revealed that awareness regarding the protection of high-risk groups significantly enhanced the willingness to vaccinate among 71% of previously unvaccinated students. Collectively, these findings suggest that both personal health perceptions and awareness of susceptibility—both for oneself and for others—play a crucial role in influencing vaccination behaviors; nonetheless, the specific pathways may vary depending on the setting and population.

This discrepancy between individuals’ improved attitudes toward vaccines and the actual rates of vaccination uptake is a paradox that will not go unnoticed. Our findings showed that individuals who perceive few benefits exhibited higher acceptability rates than those who acknowledged more significant benefits. Nonetheless, prior research has indicated positive associations between perceived benefits and vaccine acceptance (Mongeau et al., 2023; Dopelt et al., 2023). Murray and Caron offer a potential explanation for this counterintuitive observation, utilizing the framework of the HBM, which suggests that perceived susceptibility and severity may outweigh perceived benefits in the decision-making process (Murray and Caron, 2018). In other words, various moderating factors influence the relationship between perceived benefits and acceptance. As highlighted by Su and Chen, experiences and social influences are critical determinants of benefit perceptions (Su and Chen, 2023). This social dimension introduces an additional layer of complexity to vaccine acceptance, indicating that interventions aimed solely at individual knowledge or perceptions may prove inadequate.

Knowledge gaps constituted a significant barrier overall; however, the context of these specific gaps varied across the studies examined. In our study, a strong perception of benefits correlated with high barriers, whereas Hashmi et al. reported that more than half of the students acknowledged a deficiency in knowledge regarding flu vaccines (Hashmi et al., 2016). Likewise, Mallhi et al. highlighted the discrepancies in knowledge levels between healthcare and non-healthcare students (Mallhi et al., 2022). These findings suggest that traditional educational strategies may prove inadequate in addressing the multifaceted dimensions of knowledge, perception, and behavior.

### Strengths and limitations

This study presents several notable strengths that add to its contributions to understanding influenza vaccine hesitancy among university students in Saudi Arabia. A prominent strength is the use of the HBM, a theoretical framework that lends itself well to exploring the psychological determinants of vaccine uptake. This scheme provides a more nuanced perspective on factors affecting vaccine perceptions and acceptability. Finally, the analysis had a large sample size (approximately 450 university students), which increases statistical power and helps make findings more robust and generalizable, given the study population. Data collected through a well-structured and validated questionnaire and multiple rounds of revisions to data clarity further strengthen the reliability.

This study has several limitations that warrant consideration. First, the reliance on convenience sampling recruited via social media platforms introduces selection bias; this approach may overrepresent students who are more digitally active and potentially more health-conscious than the general student population, thereby limiting the generalizability of these findings to the broader national student body. Second, The sample was disproportionately composed of healthcare students due to greater access to their networks, which may limit the generalizability of the findings despite similar flu vaccination acceptance rates between healthcare and non-healthcare students. Third, Although the age category included 17-year-old participants, this reflects that some students begin university at this age. Ethical approval permitted their inclusion; however, recruiting minors online can raise consent-related considerations, which should be addressed in future studies. Second, the cross-sectional design precludes the determination of causal relationships between HBM constructs and vaccine acceptance. Furthermore, given the cross-sectional nature of the data, we cannot rule out reverse causality or cognitive dissonance mechanisms, in which unvaccinated students might retrospectively exaggerate perceived barriers or downplay perceived benefits to rationalize their behavior. Third, the use of self-reported data renders the study susceptible to social desirability bias and recall bias, potentially leading to an overestimation of positive health attitudes or inaccuracies in vaccination history. Fourth, while the HBM constructs were assessed using valid scales, the categorization of continuous Likert-scale scores into ordinal levels (Low, Moderate, High) involves arbitrary cut-offs that may lead to misclassification or the loss of nuanced data. Moreover, although gender and education were analyzed separately, small subgroup sizes limited our ability to reliably examine interaction effects (e.g., gender × education), which should be explored in future studies with larger samples. Finally, although the overall sample size was sufficient, certain subgroup analyses—such as those involving students with chronic conditions or specific marital statuses—relied on small sample sizes, which may limit the statistical reliability of those specific stratified associations.

### Future directions

The findings of this study dictate specific pathways for future research and intervention. Given our observation that high perceived benefits do not correlate with increased uptake, future interventions should move beyond general awareness campaigns that simply extol vaccine advantages. Instead, research should focus on designing and testing targeted interventions that specifically reduce logistical and psychological barriers, which were identified as the strongest predictors of hesitancy. Additionally, future studies should employ longitudinal designs to assess whether reducing these specific barriers leads to a measurable increase in vaccination rates over time. Furthermore, qualitative research is needed to explore the specific nature of the barriers reported by female students, who were found to be significantly more likely to perceive obstacles compared to their male counterparts.

## Conclusion

This study offers substantial insights into the factors influencing the intention to receive the influenza vaccine among university students in Saudi Arabia. It has been demonstrated that perceived barriers serve as the most significant predictor of vaccine acceptance. Although the advantages of vaccination are widely recognized, actual acceptance remains low, indicating that merely emphasizing these benefits may not be adequate. Future initiatives should aim to enhance vaccine uptake by addressing not only cultural barriers but also the educational barriers as perceived by students. Implementing campus-wide vaccination campaigns may also help increase vaccination rates by delivering the vaccines directly to the students instead of inviting them to visit primary healthcare centers. Furthermore, additional longitudinal research is required to assess how these perceptions evolve over time; ideally, vaccination programs should also be implemented within the region. It is imperative that a systematic approach is adopted to improve the targeting of public health efforts, thereby enhancing influenza vaccination rates and overall health within this population.

## Data Availability

The raw data supporting the conclusions of this article will be made available by the authors, without undue reservation.
